# Preexisting Atrial Fibrillation Associated with Higher Mortality in Patients with Methicillin-Resistant *Staphylococcus aureus* Bloodstream Infections: Analysis of the National Inpatient Sample

**DOI:** 10.1155/2022/8965888

**Published:** 2022-07-19

**Authors:** Timothy McCann, Michael Fatuyi, Neha Patel, Deepali B. Sharath, Anar S. Patel

**Affiliations:** ^1^Internal Medicine Department, TriHealth-Good Samaritan Hospital, 375 Dixmyth Avenue, Cincinnati 45220, Ohio, USA; ^2^Department of Infectious Diseases, TriHealth-Good Samaritan Hospital, 3219 Clifton Avenue, Cincinnati 45220, Ohio, USA

## Abstract

**Background:**

The purpose of this study was to investigate the prevalence of preexisting atrial fibrillation (AF) in patients with MRSA-BSI during a three-year period and the impact of preexisting AF on MRSA-BSI outcomes.

**Methods:**

This was a retrospective analysis performed using the National Inpatient Sample (NIS) over a three-year period (2016, 2017, 2018) for patients with MRSA-BSI as a principal diagnosis with and without preexisting AF. The primary outcome was mortality with secondary outcomes of acute coronary syndrome, cardiac arrest, cardiogenic shock, endocarditis, respiratory failure, acute kidney injury, length of stay, hospital cost, and patient charge. A univariate and multivariable logistic regression analysis estimated clinical outcomes.

**Results:**

Preexisting AF in patients with MRSA-BSI significantly increased the primary outcome of the study, mortality (1.67% vs. 0.66%, *p*=0.030) with an adjusted odds ratio (AOR) of 1.98 (95% CI, 1.1–3.7). Secondary outcomes showed increased likelihood of cardiac arrest with MRSA-BSI and AF (0.48% vs. 0.2%, *p*=0.025) with an AOR 3.59 (CI 1.18–11.0), ACS (3.44% vs. 1.21%, *p*=0.008) with an AOR of 1.81 (CI 1.16–2.80), respiratory failure (8.92% vs. 4.02%, *p*=0.045) with an AOR 1.39 (CI 1.01–1.91), prolonged LOS (6.4 vs. 5.4 days, *p*=0.0001), increased hospital cost ($13,374 vs. $11,401, *p*=0.0001), and increased overall patient charge ($50,091 vs. $43,018, *p*=0.0001). Secondary outcomes that showed statistical significance included ACS (1,497 (3.44%) vs. 113 (1.21%), *p*=0.008) with an AOR of 1.81 (CI 1.16–2.80), cardiac arrest (209 (0.48%) vs. 19 (0.2%), *p*=0.025) with an AOR 3.59 (CI 1.18–11.0), and respiratory failure (3,881 (8.92%) vs. 374 (4.02%), *p*=0.045 with an AOR 1.39 (CI 1.01–1.91).

**Conclusions:**

Preexisting AF is a significant risk factor for mortality in patients admitted for MRSA-BSI and increases risk for cardiac arrest, respiratory failure, and ACS. Considerations should focus on early treatment and source control, especially with AF given the mortality risk, increased hospitalization cost, and prolonged LOS.

## 1. Introduction

*Staphylococcus aureus* is a gram-positive coccus known to cause an array of infections including soft tissue, bone, lung, heart, kidney, surgical site infections, and bloodstream infections [1]. Categorization and treatment recommendations for infections due to *S. aureus* are based in part on resistance patterns, the most concerning being methicillin-resistant. Since the first emergence in the literature in the 1960s, the epidemiological distinction between hospital-acquired MRSA bloodstream infections (MRSA-BSI) and community-acquired MRSA-BSI has become substantially blurred as the prevalence and transmission of MRSA infections have increased [2]. At present, MRSA-BSI still accounts for a significant portion of nosocomial infections with the continued presence of community-acquired infections [3].

Despite only representing a small portion of all symptomatic *S. aureus* infections, *S. aureus* bacteremia (SAB) infections have a disproportionate impact on the burden of disease. attributable to life-threatening complications such as infective endocarditis and metastatic infections with high associated mortality rates [4, 5]. Analysis of the Emerging Infections Program (EIP) MRSA population surveillance (2005–2016) and the Premier and Cerner Electronic Health Records (2012–2017) databases found that an estimated 119,247 cases of SAB infections and 19,832 associated deaths occurred nationwide in 2017 [6]. In 2020, the Centers for Disease Control and Prevention's (CDC) National Healthcare Safety Network (NHSN) reported 8,775 MRSA-related infections across 3,500 general acute care hospitals [7]. Previous MRSA-BSI 30-day mortality estimates range from 16%–44% in general hospital populations [8–13]. Given such a wide range, it is suggested that discrepancy among MRSA reporting in the United States has likely contributed to ineffective infection control strategies [14]. Advances in antibiotic stewardship and disease prevention plans deployed in recent years have improved the incidence of MRSA infections, yet the infection remains alarmingly prevalent [15].

A key component of infection control strategies is the use of risk stratification to identify patients who are more likely to have MRSA-BSI and delineate patients more likely to suffer from complications related to this infection. Risk factors for acquiring MRSA-BSI include antibiotic use, intravenous drug use, HIV, tunneled hemodialysis catheter, and residence in long-term care facilities [16]. Unfortunately, the risk of complications and mortality associated with underlying systemic diseases in these patients has been poorly described. Thus, identifying subsets of patient populations more susceptible to infection-related complications and mortality remains a major challenge in MRSA-BSI management. Stratifying these patients based on risk can help highlight those who may benefit from close monitoring, aggressive treatment, and early intervention.

We suspect preexisting cardiac dysfunction to be a risk factor with a significant effect on mortality given the already high mortality associated with MRSA-BSI. The primary purpose of this study is to determine whether preexisting AF is a significant risk factor for mortality in patients hospitalized with MRSA-BSI. In this study, we use a well-characterized database to investigate the incidence of preexisting AF among patients admitted with MRSA-BSI. We also investigate the impact of preexisting AF on in-hospital mortality, acute coronary syndrome, cardiogenic shock, endocarditis, respiratory failure, acute kidney injury, length of stay, hospital cost, and patient charge for patients admitted with MRSA-BSI. With this analysis, we hope to build upon our understanding of the role such underlying disease may have on associated sequelae of MRSA-BSI and ultimately improve management.

## 2. Materials and Methods

### 2.1. Data Source

Data were extracted from the National In-patient Sample (NIS) between the years of 2016–2018. The Healthcare Cost Utilization Project (HCUP) was sponsored by the Agency for Healthcare Research and Quality (AHRQ), and the NIS is part of a family of databases and software tools developed by the Healthcare Cost Utilization Project (HCUP). The NIS was created and maintained by the AHRQ and is the largest publicly available all-payer inpatient database designed to produce U.S. regional and national estimates of patient utilization, access, cost, quality, and outcomes. It was designed as a stratified probability sample to be representative of all nonfederal acute care hospitals nationwide. The details of the design and description of the NIS can be found online at (https://www.hcup-us.ahrq.gov/nisoverview.jsp).

Discharge information includes patient demographics, primary payer, hospital characteristics, principal diagnosis, secondary diagnoses, and procedural diagnoses. Hospitals are stratified according to ownership/control, bed size, teaching status, urban/rural location, and geographic region. A 20% probability sample of all hospitals within each stratum is then collected. All discharges from these hospitals are recorded and weighted to ensure that they are nationally representative. Data from 48 statewide data organizations (47 states plus the District of Columbia) encompassing more than 97% of the U.S. population is included in the NIS 2016, 2017, and 2018 sampling frame.

Diagnoses are divided into two separate categories: principal diagnosis and secondary diagnoses. A principal diagnosis is the main International Classification of Diseases revision 10 (ICD-10) code for the hospitalization (I10_DX1). Secondary diagnoses include all other ICD-10 codes used other than the principal diagnosis (I10_DX2—I10D_X40). Comorbidity burden was assessed using the Charlson comorbidity index (CCI) [17]. This is a score that categorizes comorbidities based on ICD diagnosis codes and may be used to predict hospital resource use and in-hospital outcomes. All patient data in NIS are both de-identified and publicly available. Therefore, Institutional Review Board (IRB) approval was not needed.

### 2.2. Sample Selection and Study Variables

**Figure 1:**
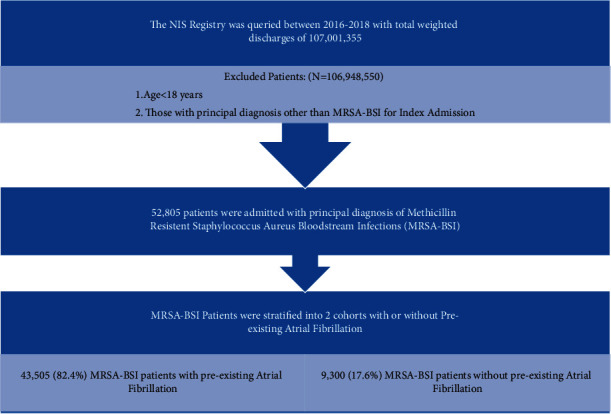
Schematic representation of the study design.

We conducted a retrospective cohort study of hospitalizations using NIS years 2016, 2017, and 2018 with a principal diagnosis of methicillin-resistant *Staphylococcus aureus* Bloodstream Infection (MRSA-BSI) with secondary diagnoses of with and without AF in acute care hospitals across the United States. Hospitalizations were selected from the NIS database found online at https://www.hcup-us.ahrq.gov. The study population consisted of all inpatient hospitalizations recorded in the NIS 2016, 2017, and 2018 for patients 18 years old and above meeting our diagnostic criteria (Figure 1). Study variables included age, gender, race, and hospital characteristics including teaching vs. nonteaching; hospital bed size (small, medium, and large); hospital region (northeast, midwest, south, and west); insurance (Medicare, Medicaid, private, and others); median annual income expected for patient's Zone Improvement Plan (ZIP) code; medical comorbidities; and primary and secondary outcomes (described further below).

We used the following ICD-10 codes to identify principal and secondary diagnoses: MRSA-BSI ICD 10 codes, B9562-R7881 and Atrial Fibrillation, I480, I481, I482, I4891 (Table A1). We studied baseline characteristics, inpatient mortality predictors, and outcomes (primary and secondary) for MRSA-BSI hospitalizations with preexisting AF vs. MRSA-BSI hospitalizations without preexisting AF.

### 2.3. Outcomes Measured

The primary outcome was inpatient mortality among patients principally admitted for MRSA-BSI with vs. without a secondary diagnosis of preexisting AF. Secondary outcomes evaluated were acute coronary syndrome (ACS), cardiac arrest, cardiogenic shock, endocarditis, respiratory failure, acute kidney injury (AKI), length of stay (LOS), hospital cost, and patient charges for MRSA-BSI hospitalizations with preexisting AF vs. without preexisting AF.

Baseline patient characteristics included demographics (age, sex, race), primary expected payer, median household income for the patient's ZIP code, hospital characteristics (teaching vs. nonteaching), bed size (small, medium, and large), hospital region (northeast, midwest, south, and west), Charlson comorbidities, as defined by the AHRQ, which include congestive heart failure, myocardial infarction, peripheral vascular disease, cerebrovascular disease, dementia, chronic pulmonary disease, rheumatic disease, peptic ulcer disease, liver disease, diabetes without chronic complication, diabetes with chronic complication, hemiplegia or paraplegia, renal disease, any malignancy (solid, leukemia, lymphoma except skin malignancy), metastatic solid tumor, and HIV/AIDS.

### 2.4. Statistical Analysis

Analyses were performed using STATA (Statistics and Data Science), version 17.0 NP-Parallel Edition (Stata Corp, Texas, USA). Proportions were compared using the Fisher exact test, and continuous variables were compared using the independent Student's *t*-test. A regression model was applied for our analysis. For binary, dichotomous, or categorical variables, logistic regression was used. Poisson regression was used for discrete variables due to not normal variable distribution. Linear regression was used for continuous variables. Univariate logistic regression, linear regression, and Poisson regression model analyses were used for all unadjusted outcome variables. A univariate model was used to calculate unadjusted odds ratios (ORs) for the primary and secondary outcomes. Univariable and multivariable analysis for the predictors of mortality was performed with a multivariable linear Cox proportional hazards regression model that was stratified for our study.

Multivariable logistic, linear, and Poisson regression was used to calculate adjusted odds ratios (ORs) for the primary and secondary outcomes. Multiple imputations were used for less than 1 percent of missing data for the race. All variables with *p* values <0.1 with our univariate analysis were included in a multivariable logistic regression model. All *p* values were two-sided, and a *p* value <0.05 was considered significant in the multivariable analysis. The severity of comorbidities was quantified using the Charlson comorbidity index. The Charlson comorbidity index was first developed in 1987 by Mary Charlson and colleagues as a weighted index to predict risk of death [18]. The Charlson comorbidity index was used to adjust for comorbidity burden for the primary and secondary outcomes. The Charlson comorbidity index (CCI) score: 0 = no comorbidities, 1 = low comorbidity burden, 2 = moderate comorbidity burden, and 3 or greater = high comorbidity burden. The CCI has been used extensively in clinical research; it is commonly used to assess mortality risk, and it is supported by extensive validity evidence [19]. Higher scores have been associated with mortality or greater healthcare resource use [20]. The comorbidity score was then calculated for each patient by summing the individual weights of all comorbidities. Weighted estimates were calculated by applying discharge weight to the unweighted discharge records. Weighted estimates were used for all statistical analyses.

Covariates included in the adjusted models were age, sex, race, insurance provider, hospital characteristics, hospital regions, household earnings, nicotine use, baseline oxygen use, and Charlson comorbidity index, which include congestive heart failure, myocardial infarction, peripheral vascular disease, cerebrovascular disease, dementia, chronic pulmonary disease, rheumatic disease, peptic ulcer disease, liver disease, diabetes without chronic complication, diabetes with chronic complication, hemiplegia or paraplegia, renal disease, any malignancy (solid, leukemia, lymphoma except skin malignancy), metastatic solid tumor, and HIV/AIDS.

## 3. Results

### 3.1. Patient Characteristics

A total of 52,805 patients were infected with MRSA-BSI during the three-year timeframe evaluated by our study with 9,300 (17.61%) having preexisting AF. The AF versus non-AF cohorts had a median interquartile range (IQR) age of 75.3 years (CI 74.8–75.9) vs. 59.6 years (CI 59.2–60.0). Male sex was predominant (28,409 (65.3%) vs. 5,087 (54.7%)). The predominant ethnicity was white (36,109 (83%) vs. 6,445 (69.2%)) (Table 1).

Statistically significant comorbidities included coronary artery disease (CAD), congestive heart failure (CHF), previous myocardial infarction (MI), chronic kidney disease (CKD), dyslipidemia, nicotine use, chronic liver disease, peripheral vascular disease (PVD), obesity, pulmonary hypertension, previous coronary artery bypass graph (CABG), previous pacemaker, previous defibrillator, uncomplicated diabetes, complicated diabetes, previous percutaneous intervention (PCI), carotid artery disease, electrolyte abnormalities, COPD, oxygen use, frailty, dementia, and long-term anticoagulation use (Table 1).

CCI evaluation that reached statistical significance (*p* < 0.0001) included a distribution of 0–2 (24,624 (56.6%) vs. 6,538 (70.3%)), 3–5 (3,179 (7.2%) vs. 1,051 (11.3%)), >6 (15,749 (36.2%) vs. 1,711 (18.4%)) (Table 1).

### 3.2. Outcomes

MRSA-BSI with preexisting AF significantly increased the primary outcome of the study, mortality (727 (1.67%) vs. 61 0.66%), *p*=0.030) with an adjusted odds ratio (AOR) of 1.98 with a 95% CI [1.1–3.7] (Table 2).

A multivariable Cox proportional hazards regression analysis for predictors of in-hospital mortality among the patients with MRSA-BSI and AF showed adjusted hazard ratios (HRs) associated with the following variables: age<65 (HR = 1.03, 95% CI: 1.01–1.06, *p*=0.002), protein calorie malnutrition (HR = 2.6, 95% CI: 1.35–4.86, *p*=0.004), CCI ≥6 (HR = 2.3, 95% CI: 1.25–4.16, *p*=0.007), end-stage liver disease (HR = 2.50, 95% CI: 1.4–4.70, *p*=0.003), acute coronary syndrome (HR = 3.5, 95% CI: 1.43–8.68, *p*=0.006), ST-elevation myocardial infarction (STEMI) (HR = 6.6, 95% CI: 2.34–18.27, *p* < 0.0001), cardiogenic shock (HR = 3.8, 95% CI: 1.76–8.28, *p*=0.001), acute respiratory failure (HR = 6.20, 95% CI: 3.2–11.68, *p* < 0.0001), and septic shock (HR = 7, 95% CI: 3.70–11.70, *p* < 0.0001) (Table 3).

Secondary outcomes that showed statistical significance included ACS (1,497 (3.44%) vs. 113 (1.21%), *p*=0.008) with an AOR of 1.81 (CI 1.16–2.80); cardiac arrest (209 (0.48%) vs. 19 (0.2%), *p*=0.025) with an AOR 3.59 (CI 1.18–11.0); and respiratory failure (3,881 (8.92%) vs. 374 (4.02%), *p*=0.045 with an AOR 1.39 (CI 1.01–1.91) (Table 2).

For preexisting AF and MRSA-BSI, length of stay (LOS) was longer (6.4 vs. 5.4 days, *p*=0.0001) with an AOR 1.14 (CI 1.1–1.21), increased hospital cost ($13,374 vs. $11,401, *p*=0.0001) with an AOR 1.13 (CI 1.06–1.20), and increased overall patient charge ($50,091 vs. $43,018, *p*=0.0001) with an AOR 1.17 (CI 1.08–1.26) (Table 2).

## 4. Discussion

The aim of this study was to identify and quantify mortality risk associated with AF in MRSA-BSI. Early identification of patients at increased risk of mortality who present with MRSA-BSI can help guide urgent and aggressive management. In this large observational retrospective cohort study, we found 52,805 patients with MRSA-BSI for the three years observed (2016–2018), and among those, 43,505 (82.4%) had preexisting AF. Patients with MRSA-BSI and AF were more likely to have ACS, cardiac arrest, and respiratory failure in addition to increased LOS, hospital cost, and patient charge compared to those without AF (Table 2).

Patients with preexisting AF were more likely to have a CCI ≥6 in addition to the increased presence of cardiac abnormalities including CAD, CHF, previous MI, previous CABG, previous pacemaker, previous defibrillator, and previous PCI. Atrial fibrillation is by definition a type of heart failure, so increased presences of other cardiac abnormalities follow. Common related medical issues that were also at increased presence including CKD, dyslipidemia, long-term use of anticoagulation, PVD, obesity, pulmonary hypertension, oxygen use, frailty, COPD, dementia, previous stroke, uncomplicated diabetes, and complicated diabetes. There was decreased use of aspirin in the AF cohort, likely related to increased use of anticoagulants although distinguishing type, amount, and adherence with these data are not possible (Table 1).

Preexisting AF was associated with a significant increase in the primary outcome of the study, showing a two-fold increase in mortality associated with AF in MRSA-BSI (AOR = 1.98). They were also more likely to have ACS (AOR = 1.81), cardiac arrest (AOR = 3.59), and respiratory failure (AOR = 1.39) compared to patients without AF. These data align with our hypothesis that the infectious burden of MRSA on a circulatory system with underlying cardiac dysfunction leads to increased mortality. The analysis of hazard ratios for predictors of in-hospital mortality in MRSA-BSI with AF showed increased mortality risk from 7.7-fold to 2.3-fold with septic shock (aHR = 7.7), STEMI (aHR = 6.6), acute respiratory failure (aHR = 6.2), cardiogenic shock (aHR = 3.8), ACS (aHR = 3.5), protein calorie malnutrition (aHR = 2.6), end-stage liver disease (aHR = 2.5), and CCI ≥6 (aHR = 2.3) (Table 3). These hazard ratios suggest the presence of respiratory, cardiac, or septic compromise led to a high likelihood of mortality in this subset of patients.

This is the first known study to evaluate patients with MRSA-BSI and the effect of preexisting AF. Patients with AF have been reported to demonstrate a 3.67-fold higher risk of all-cause death than an age- and sex-matched general population [21]. Early data from the Framingham Heart Study place a 1.5–1.9-fold mortality risk from AF [22]. Newer studies have found reduction in the hazard ratios associated with AF over time as well as decreasing trends in the association between AF and noncardiovascular death, but not in relation to cardiovascular disease [23]. No study to our knowledge has assessed MRSA-BSI and AF.

Multiple prior studies have evaluated predictive risk factors for mortality in MRSA-BSI. These previous studies, however, focus primarily on initial infection source, locations of acquisition, and appropriate treatments [10, 24, 25]. The choice of appropriate empiric antibiotics has also been shown to play a role in treatment success [9, 12, 26]. Other studies have evaluated risk factors and mortality data in MRSA-BSI and found mortality risk surrounding age, co-occurring neoplasm, and duration of hospital stay [11, 13]. Additional risk factors for mortality with MRSA-BSI include hematologic malignancy, hematopoietic stem cell transplantation, community-onset infection, secondary bloodstream infection, high minimum inhibitory concentration (MIC) towards vancomycin (≥2.0 *μ*g/mL), mechanical ventilation, and late switch to an alternative therapy (≥4 days after treatment failure) [27]. A recent study from Korea found severe sepsis and septic shock as statistically significant independent risk factors associated with early mortality [28]. Our study demonstrated a demonstrably increased risk of mortality in patients who experienced septic shock with preexisting AF in MRSA-BSI. While many of the risk factors evaluated in the literature play an extensive role and effect on mortality in MRSA-BSI, our study further explored the specific risk of cardiac dysfunction in AF on outcomes related to MRSA-BSI. Quantifying underlying disease risk is a necessary foundational component to risk stratifying MRSA-BSI patients.

There are limitations to our study. First, a causal relationship cannot be established as the study is retrospective in nature. We were unable to quantify or account for confounding variables that were unmeasured. Furthermore, the NIS-stratified probability sample, designed to be representative of all nonfederal acute care hospitals nationwide, accounts for only 20% of all the hospital admissions in the United States. The NIS sample is also limited in terms of data collection as it is based primarily on ICD codes used for billing purposes. The HCUP quality control measures are intended to minimize this possibility. Further analysis of the severity of comorbid conditions was not possible as data are limited to only ICD code diagnosis. Nevertheless, the NIS database is extremely large and weighted to reflect the national average. Therefore, while these limitations do exist, they are compensated by the advantage of large patient volumes and ability to evaluate data on a national scale.

## 5. Conclusion

To our knowledge, this is the largest study ever published evaluating preexisting atrial fibrillation as a risk factor for mortality in patients admitted for MRSA-BSI. The preexistence of AF in MRSA-BSI patients poses a significant increased risk of mortality. It also possesses a statistically significant increased risk of cardiac arrest, respiratory failure, and ACS. Considerations should focus on early treatment and source control especially in patients with AF given the mortality risk, increased hospitalization cost, patient charge, and prolonged LOS.

## Figures and Tables

**Table 1:** Study population patient characteristics stratified by MRSA-BSI with and without AF.

Characteristics	MRSA-BSI with AF (*N*, %)	MRSA-BSI without AF (*N*, %)	*p* value
(Total *N* = 52,805)	43,505 (82%)	9,300 (18%)	
Mean age, in years (IQR)	75.3 (IQR = 74.8–75.9)	59.6 (IQR = 59.2–60.03)	<0.0001
18–44 years	870 (2%)	2,074 (22.3%)	
45–64 years	6,0004 (13.8%)	3,143 (33.8%)	
≥65 years	36,631 (84.2%)	4,083 (43.9%)	
Race			<0.0001
White	36,109 (83%)	6,445 (69.2%)	
Black	3,437 (7.9%)	1,404 (15.1%)	
Hispanic	2,045 (4.7%)	911 (9.8%)	
Others	1,914 (4.4%)	549 (5.9%)	
Male	28,409 (65.3%)	5,087 (54.7%)	<0.0001
Female	15,096 (34.7%)	4213 (45.3%)	<0.0001
Insurance			<0.0001
Medicare	35,761 (82.2%)	4808 (51.7%)	
Medicaid	2,001 (4.6%)	1,832 (19.7%)	
Private insurance including HMO	4742 (10.9%)	1972 (21.2%)	
Other/self-pay/uninsured	1,001 (2.3%)	707 (7.4%)	
Number of hospital beds			0.01
Small	10,006 (23%)	1981 (21.3%)	
Medium	12,442 (28.4%)	2,437 (26.2%)	
Large	21,153 (48.6%)	4,883 (52.5%)	
Hospital teaching status			0.001
Nonteaching hospital	15,488 (35.6%)	2,892 (31.1%)	
Teaching hospital	28,017 (64.4%)	6,408 (68.9%)	
Hospital region			0.21
Northeast	10,403 (23.9%)	2,111 (22.7%)	
Midwest	10,398 (23.9%)	2,083 (22.4%)	
South	15,096 (34.7%)	3,404 (36.6%)	
West	7,613 (17.5%)	1,702 (18.3%)	
Median annual income expected for patient's ZIP code, USD			<0.0001
$1—$45,999	10,354 (23.8%)	2,781 (29.9%)	
$46,000—$58,999	11,833 (27.2%)	2,585 (27.8%)	
$59,000—$78,999	10,963 (25.2%)	2,176 (23.4%)	
≥$79,000	10,354 (23.8%)	1,758 (18.9%)	
Comorbidities			
Coronary artery disease	18,185 (41.8%)	1,683 (18.1%)	<0.0001
Congestive heart failure	19,490 (44.8%)	1,097 (11.8%)	<0.0001
Previous MI	8,788 (20.2%)	781 (8.5%)	<0.0001
Chronic kidney disease	16,967 (39%)	2,027 (21.8%)	<0.0001
Hypertension	15,227 (35%)	3,432 (36.9%)	0.1
Previous stroke	261 (0.6%)	28 (0.3%)	0.03
Dyslipidemia	21,970 (50.5%)	2,874 (30.9%)	<0.0001
Nicotine use	14,912 (34%)	3,581 (38.5%)	0.0003
Chronic liver disease	2,784 (6.4%)	809 (8.7%)	0.0009
Peripheral vascular disease	2,5667 (5.9%)	270 (2.9%)	<0.0001
Obesity	7,526 (17.3%)	1,228 (13.2%)	<0.0001
Anemia	16,184 (37.2%)	3,339 (35.9%)	0.27
Pulmonary hypertension	3,785 (8.7%)	233 (2.5%)	<0.0001
Previous CABG	5,569 (12.8%)	428 (4.6%)	<0.0001
Previous pacemaker	5,960 (13.7%)	233 (2.5%)	<0.0001
Previous defibrillator	3,306 (7.6%)	140 (1.5%)	<0.0001
Uncomplicated diabetes	17,881 (41.1%)	2,957 (31.8%)	<0.0001
Complicated diabetes	11,442 (26.3%)	1,609 (17.3%)	<0.0001
Previous PCI	479 (1.1%)	56 (0.6%)	0.006
Carotid artery disease	609 (1.4%)	52 (0.56%)	<0.0001
Electrolyte abnormalities	13,443 (30.9%)	2,660 (28.6%)	0.047
Dialysis dependent	3,485 (8.01%)	716 (7.7%)	0.64
COPD	7,526 (17.3%)	1,014 (10.9%)	<0.0001
Oxygen use	1,740 (4%)	177 (1.9%)	<0.0001
Protein energy malnutrition	3,654 (8.4%)	707 (7.6%)	0.24
Frailty	130 (0.3%)	74 (0.08%)	0.007
Dementia	5,308 (12.2%)	614 (6.6%)	<0.0001
Long term anticoagulation use	16,967 (39%)	521 (5.6%)	<0.0001
Aspirin use	6,047 (13.9%)	1,730 (18.6%)	<0.0001
Charlson comorbidity index			<0.0001
0–2	24,624 (56.6%)	6,538 (70.3%)	
3–5	3,179 (7.2%)	1,051 (11.3%)	
≥6	15,749 (36.2%)	1,711 (18.4%)	

**Table 2:** Primary and secondary outcomes associated with MRSA-BSI with and without AF.

Outcome	MRSA-BSI with AF,*N* = 43,505	MRSA-BSI without AF,*N* = 9,300	Adjusted OR	Adjusted IRR	95% CI	*p* value
Mortality	727 (1.67%)	61 (0.66%)	1.98	—	1.1–3.7	0.030
Acute coronary syndrome	1,497 (3.44%)	113 (1.21%)	1.81	—	1.16–2.80	0.008
Cardiac arrest	209 (0.48%)	19 (0.2%)	3.59	—	1.18–11.0	0.025
Cardiogenic shock	96 (0.22%)	5 (0.05%)	3.7	—	0.2–13.99	0.230
Endocarditis	844 (1.94%)	143 (1.54%)	1.11	—	0.63–1.96	0.725
Respiratory failure	3,881 (8.92%)	374 (4.02%)	1.39	—	1.01–1.91	0.045
Acute kidney injury	9,776 (22.47%)	1,376 (14.78%)	1.17	—	0.96–1.42	0.116
Length of stay	6.4 days	5.4 days	—	1.14	1.1–1.21	0.0001
Hospital cost	$13,374	$11,401	—	1.13	1.06–1.20	0.0001
Patient charge	$50,091	$43,018	—	1.17	1.08–1.26	0.0001

OR *=* odds ratio; IRR *=* *i*ncidence rate ratio; CI *=* confidence interval.^*∗*^Adjusted for age, sex, race, hospital characteristics (teaching status, hospital regions), insurance, household earnings, nicotine use, oxygen use, myocardial infarction, and Charlson comorbidity index.

**Table 3:** Multivariable cox proportional hazards regression analysis for predictors of in-hospital mortality among MRSA-BSI patients with AF.

Variables	aHR	Lower CI	Upper CI	*p* value
Age (ref =<65)	1.03	1.01	1.06	0.002
Protein calorie malnutrition	2.6	1.35	4.86	0.004
Charlson comorbidity index ≥6 ^†^(ref: =<5)	2.3	1.25	4.16	0.007
End-stage liver disease	2.5	1.4	4.7	0.003
Acute coronary syndrome	3.5	1.43	8.68	0.006
STEMI	6.6	2.34	18.27	<0.0001
Cardiogenic shock	3.8	1.76	8.28	0.001
Acute respiratory failure	6.2	3.2	11.68	<0.0001
Septic shock	7.7	3.7	11.7	<0.0001

## Data Availability

Data were extracted from the National In-patient Sample (NIS) between the years of 2016–2018. The details of the design and description of the NIS can be found online at https://www.hcup-us.ahrq.gov/nisoverview.jsp.

## Abbreviations

AF: Atrial fibrillation
MRSA-BSI: Methicillin-resistant *Staphylococcus aureus* bloodstream infection
ACS: Acute coronary syndrome
AKI: Acute kidney injury
LOS: Length of stay
AOR: Adjusted odds ratio
SAB: *Staphylococcus aureus* bacteremia
EIP: Emerging infections program
MRSA: Methicillin-resistant *Staphylococcus aureus*
CDC: Centers for Disease Control
NHSN: National Healthcare Safety Network
HIV: Human immunodeficiency virus
NIS: National Inpatient Sample
HCUP: Healthcare Cost Utilization Project
AHRQ: Agency for Healthcare Research and Quality
ICD: International Classification of Diseases
IRB: Institutional Review Board
MRSA-B: Methicillin-resistant *Staphylococcus aureus* bacteremia
ZIP: Zone improvement plan
AIDS: Acquired immunodeficiency syndrome
STATA: Statistics and data science
ORs: Odds ratios
CCI: Charlson comorbidity index
IQR: Interquartile range
CI: Confidence interval
CAD: Coronary artery disease
CHF: Congestive heart failure
MI: Myocardial infarction
CKD: Chronic kidney disease
PVD: Peripheral vascular disease
CABG: Coronary artery bypass graft
PCI: Percutaneous intervention
AOR: Adjusted odds ratio
HR: Hazard ratio
STEMI: ST-elevation myocardial infarction
MIC: Minimum inhibitory concentration
IRB: Institutional Review Board
*μ*g/mL: Microgram per milliliter.

## Additional Points

Timothy McCann and Michael Fatuyi are internal medicine residents at TriHealth-Good Samaritan Hospital in Cincinnati, Ohio.

## Supplementary Materials

Supplementary Materials Supplementary Table A1 is an all-inclusive list of ICD-10 codes used to extract data within the study for diagnosis, comorbidities, and hospital outcomes.
