# Supplementary material for: Preexisting Atrial Fibrillation Associated with Higher Mortality in Patients with Methicillin-Resistant Staphylococcus aureus Bloodstream Infections: Analysis of the National Inpatient Sample
Source: Interdiscip Perspect Infect Dis. 2022 Jul 19;2022:8965888. doi: 10.1155/2022/8965888 (PMC9325627; doi:10.1155/2022/8965888)
Supplement: Supplementary Materials — Supplementary Table A1 is an all-inclusive list of ICD-10 codes used to extract data within the study for diagnosis, comorbidities, and hospital outcomes. [file 8965888.f1.docx]

Supplementary Table A.1. ICD-10 codes Used

|  | ICD-10 codes |
| --- | --- |
| **Diagnosis codes (primary and secondary)** |  |
| MRSA bloodstream Infection | B9562-R7881 |
| Atrial fibrillation | I480, I481, I482, I4891 |
| **Comorbidities** |  |
| Coronary artery disease | I25 |
| Dyslipidemia | E78 |
| Old or previous MI | I252 |
| Old or previous PCI | Z9861 |
| Old or previous CABG | Z951 |
| Old pacemaker | Z950 |
| Protein energy malnutrition | E45, E46, E42, E43, E44, E440, E441 |
| Frailty/Age related debility | R54 |
| Chronic obstructive pulmonary disease | J41, J42, J43, J44 |
| Baseline oxygen use | Z9981 |
| Carotid artery disease | I652 |
| Old or previous stroke | I63 |
| Hypertension | I10 |
| Peripheral vascular disease | I739, I738 |
| Diabetes mellitus Type 1&2 | E10, E11 |
| Complicated diabetes mellitus Type 1&2 | E119 |
| Obesity | E660, E6601, E6609, E661, E662, E668, E669, Z6831, Z684. |
| Congestive heart failure | I50 |
| Chronic kidney disease | N18 |
| Dialysis dependent | Z992 |
| Liver disease | K70, K71, K72, K73, K74, K75, K76, K77 |
| Electrolyte derangement | E870, E871, E872, E873, E874, E875, E876 |
| Oxygen dependence | Z9981 |
| Nicotine Use | Z87891, F172 |
| Anemia | D50, D51, D52, D53, D55, D56, D57, D58, D59, D60, D61, D62, D63, D64 |
| Pulmonary Hypertension | I270, I272 |
| Dementia | F01, F02, F03 |
| Long term anticoagulation | Z7901, Z7902 |
| Long term aspirin | Z7982 |
| **Hospital outcomes** | |
| Endocarditis | I33 |
| Acute coronary syndrome | I240, I248, I249, I200, I214, I201, I208, I209, I2101, I2102, I2109, I210, I211, I212, I213, I214, I219, I211, I22, I210 |
| Cardiogenic shock | R570 |
| Cardiac arrest | I46 |
| Acute respiratory failure | J960, J962, J969 |
| Invasive mechanical ventilator | Z9911 |
| Acute kidney injury | N17 |
| Severe sepsis with septic shock and shock, unspecified | R6521, R6520, R579 |
| Abbreviations: MI: Myocardial infarction, PCI: percutaneous coronary intervention, CABG: Coronary artery bypass graft | |
